# Lower trapezius transfer with Achilles tendon augmentation: indication and clinical results

**DOI:** 10.1007/s11678-018-0489-6

**Published:** 2018-11-07

**Authors:** William R. Aibinder, Bassem T. Elhassan

**Affiliations:** Department of Orthopedic Surgery, Mayo Clinic, 200 First Street SW, 55905 Rochester, MN USA

**Keywords:** External rotation, Tendon transfer, Pseudoparalysis, Shoulder injuries, Rotator cuff tear, Außenrotation, Sehnentransfer, Pseudoparalyse, Schulterläsionen, Rotatorenmanschettenruptur

## Abstract

Lower trapezius tendon transfer with Achilles tendon allograft augmentation may be used to treat patients with lack of active external rotation following shoulder paralysis or massive irreparable posterosuperior rotator cuff tears. In the setting of shoulder paralysis, the integrity of the ipsilateral lower trapezius may be compromised. In this instance, the contralateral lower trapezius may be used with reasonable results. In the setting of irreparable rotator cuff tears, the procedure may be performed through an open or arthroscopically assisted technique. The latter avoids the need for an acromial osteotomy and risk of nonunion associated with repair of the osteotomy. Both are effective in reversing pseudoparesis or pseudoparalysis. Advanced degenerative changes have an effect on outcomes, resulting in less pain improvement, decreased range of motion, and greater need for reoperation with conversion to reverse total shoulder arthroplasty. Nonetheless, the lower trapezius tendon transfer is an effective option for restoring active external rotation with relatively consistent results.

## Zusammenfassung

Der Transfer der unteren Trapeziussehne mit Augmentation durch ein Achillessehnen-Allotransplantat kann zur Therapie von Patienten mit mangelnder aktiver Außenrotation nach Schulterlähmung oder massiven irreparablen posterosuperioren Rotatorenmanschettenrupturen eingesetzt werden. Bei Vorliegen einer Schulterlähmung kann die Integrität des ipsilateralen unteren M. trapezius beeinträchtigt sein. In diesem Fall kann der kontralaterale M. trapezius mit akzeptablen Ergebnissen verwendet werden. Liegt eine irreparable Rotatorenmanschettenruptur vor, so kann die Therapie über eine offene Operation oder arthroskopisch erfolgen. Bei Letzterer werden die Notwendigkeit einer akromialen Osteotomie und das Risiko einer Pseudarthrose bei deren Heilung vermieden. Beide Verfahren eignen sich zur Beseitigung einer Pseudoparese oder Pseudoparalyse. Fortgeschrittene degenerative Veränderungen wirken sich auf das Ergebnis aus, führen zu einer geringeren Schmerzlinderung, vermindertem Bewegungsumfang und höherem Bedarf an Reoperationen mit Konversion zur reversen totalen Schulterarthroplastik. Trotzdem stellt der Transfer der unteren Trapeziussehne eine wirksame Option zur Wiederherstellung der aktiven Außenrotation mit relativ konstanten Ergebnissen dar.

Pseudoparesis or pseudoparalysis is debilitating in young patients desiring an active lifestyle. This presentation is often associated with irreparable rotator cuff tears with no or minimal degenerative changes. Attempts at surgical repair often result in a high re-tear rate [9]. Although reverse total shoulder arthroplasty results in effective pain relief, this option is often reserved for patients with degenerative changes and a less active lifestyle [17]. Tendon transfer surgery has become a more accepted treatment option for irreparable rotator cuff tears in patients desiring greater postoperative function, particularly strength and range of motion [8, 10, 13, 14]. Specifically, the lower trapezius tendon transfer with Achilles tendon allograft augmentation is indicated for massive irreparable posterosuperior rotator cuff tears in patients with limited degenerative changes as well as in patients with paralytic shoulders following nerve injuries.

## Indications

**Fig. 1:**
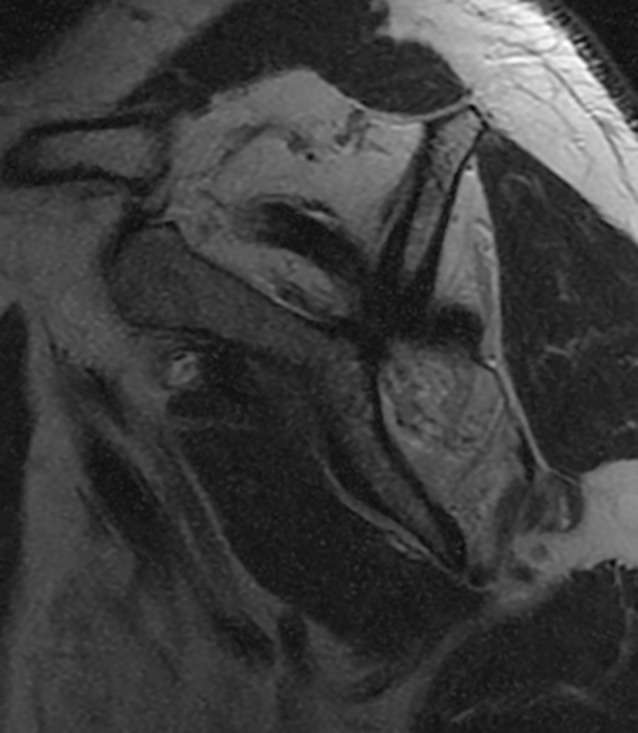
Sagittal T1-weighted magnetic resonance image demonstrating significant atrophy of the supraspinatus and infraspinatus tendons, representative of an irreparable rotator cuff tear

The lower trapezius tendon transfer with Achilles tendon allograft augmentation is indicated for massive irreparable posterosuperior rotator cuff tears and for paralytic shoulders lacking active external rotation (Fig. 1).

Absolute contraindications include active soft tissue infection or trapezius muscle paralysis, which may occur following trauma or prior to transfer of the spinal accessory nerve in treatment of brachial plexus injuries. Relative contraindications include advanced glenohumeral degenerative changes or inability of the patient to follow postoperative instructions.

Nonetheless, the basis of the procedure relies on several key principles of the glenohumeral articulation and rotator cuff balance. The glenohumeral articulation is an inherently unstable joint and relies heavily on the dynamic stabilization of the rotator cuff musculature [11]. In order for this to occur effectively, the transverse and coronal force couples must be intact and balanced [3]. Thus, the anterior subscapularis must be balanced relative to the posterior portion of the infraspinatus and teres minor; while the superior force provided by the deltoid must be balanced relative to the rotator cuff musculature inferior to the humeral head equator. This serves to maintain a concentric joint that facilitates shoulder function.

In the setting of shoulder paralysis, the static effect of the shoulder musculature maintains the glenohumeral articulation, but the shoulder lacks power to function. These patients are often limited by the lack of active external rotation, which has been shown to be important in many activities of daily living [2]. Thus, a lower trapezius tendon transfer in this setting aims to restore active external rotation and provide function.

In the setting of a massive irreparable posterosuperior rotator cuff tear, the force couples are often imbalanced and patients present with not only lack of function, but also significant pain and debility [14]. A lower trapezius tendon transfer in this setting aims to primarily relieve pain, but also to provide strength and function. The anterior portion of the transverse couple must be intact, or if torn, must be recreated by tendon transfer or repair. Ultimately, the goal is to reverse pseudoparesis or pseudoparalysis of the shoulder.

The biomechanical rationale for the lower trapezius tendon transfer is based on two key studies [12, 15]. Hartzler et al. demonstrated that the external rotation moment arm with the arm at the side was greatest with the lower trapezius transfer. Omid et al. demonstrated that the native glenohumeral kinematics and joint reactive forces were most closely recreated when the lower trapezius transfer was performed. In essence, the osseous concentricity of the joint is best recreated with the lower trapezius tendon transfer allowing for the greatest improvements in range of motion. Additionally, it appears that the trapezius muscle demonstrates electrical recruitment during active external rotation.

**Fig. 2:**
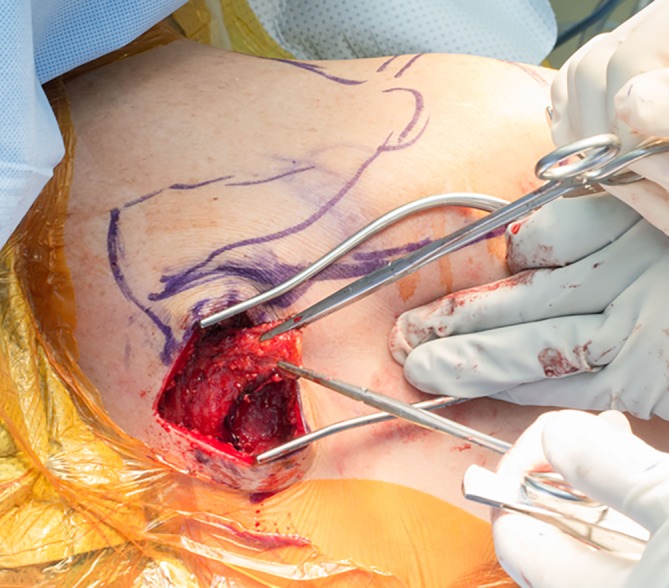
Harvesting of the lower trapezius tendon through a horizontal incision along the medial aspect of the scapular spine. Note the triangular shape of the tendon

**Fig. 3:**
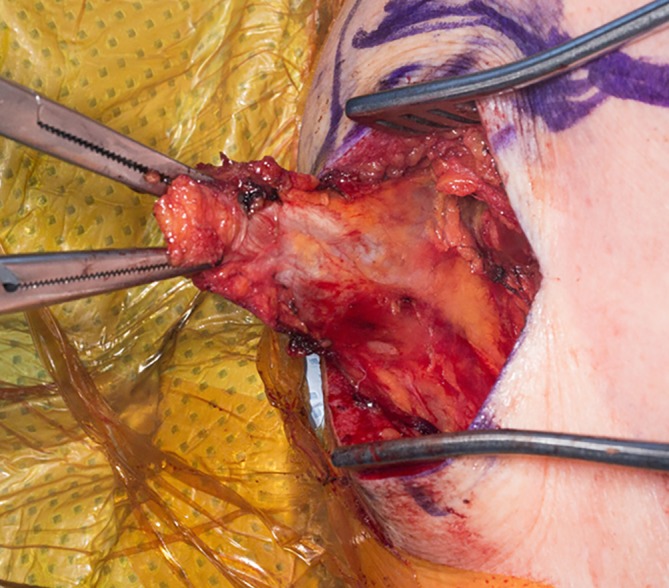
Medial dissection on the under-surface of the tendon allows for maximal excursion and interdigitation with the Achilles tendon allograft

Achilles tendon allograft augmentation is required in all cases of irreparable rotator cuff tears owing to the short excursion of the lower trapezius tendon (Figs. 2 and 3). Augmentation, however, is not indicated in all cases of shoulder paralysis as long as the integrity and caliber of the infraspinatus tendon are adequate.

## Clinical results

### Paralytic shoulder

Promising results have reported of lower trapezius tendon transfer for an indication of shoulder paralysis with absent active external rotation. Although there is a paucity of data, several case reports and case series exist utilizing ipsilateral lower trapezius tendon transfer for restoration of active external rotation in brachial plexus injuries (Table 1). Bertelli reported on seven adult patients who regained a mean of 104° of active external rotation as measured from the abdomen [1]. Elhassan et al. reported improvements in active external rotation in 46 patients [4, 5].

**Table 1:** Summary of recent studies evaluating lower trapezius tendon transfer for restoration of active external rotation

	Indication	Technique	Patients	Final External Rotation
Bertelli (2011; [1])	Shoulder paralysis	Open	7	104° (from abdomen)
Satbhai et al. (2014; [16])	Shoulder paralysis	Contralateral open	3	97° (from abdomen)
Elhassan et al. (2016; [7])	Shoulder paralysis	Contralateral open	12	110° (from abdomen)
Elhassan et al. (2016; [8])	Irreparable rotator cuff tear	Open	33	50° (from neutral)
Elhassan et al. (unpublished)	Irreparable rotator cuff tear	Arthroscopically assisted	37	47° (from neutral)

In many cases of shoulder paralysis, however, the ipsilateral trapezius is often compromised owing to the inciting trauma or because of initiating reconstructive efforts using transfer of the spinal accessory nerve. Thus, several authors have used the contralateral lower trapezius muscle transfer to restore active external rotation. Satbhai et al. reported on three cases of shoulder paralysis treated with a contralateral lower trapezius tendon transfer [16]. Active external rotation improved in all cases with a mean of 97° of motion. Additionally, the authors noted significant improvements in functional outcomes. Elhassan et al. reported on 12 patients undergoing a contralateral lower trapezius tendon transfer [7]. Active external rotation improved in ten (83%) patients with a mean of 110° of motion from the abdomen. In the two failures, one patient underwent plication of the transfer following a trauma, while the other refused further treatment. These early reports demonstrate promising early outcomes. The latter study did not identify any effects of the transfer on shoulder function of the donor side.

### Irreparable posterosuperior rotator cuff tear

Open lower trapezius tendon transfer surgery for irreparable posterosuperior rotator cuff tears has been shown to result in significant improvements in pain and range of motion in 32 of 33 patients a mean follow-up of 47 months [8]. Most noteworthy is restoration of strength and range of motion. An external rotation lag sign, which was present in 82% of patients, resolved universally. All patients had a minimum of grade 4 out of 5 muscle strength on manual external rotation strength testing. External rotation pseudoparesis or pseudoparalysis resolved in all cases. Several complications have also been reported. Four patients were noted to have a seroma, which was successfully treated conservatively. One patient required a glenohumeral arthrodesis for a persistent infection. Another patient was noted to have failure of the transfer following a fall.

The open technique requires an acromial osteotomy to expose the humeral head. Radiographically, 24% of patients demonstrated incomplete healing, which did not affect outcomes. Nonetheless, a nonunion of the acromial osteotomy is worrisome.

**Fig. 4:**
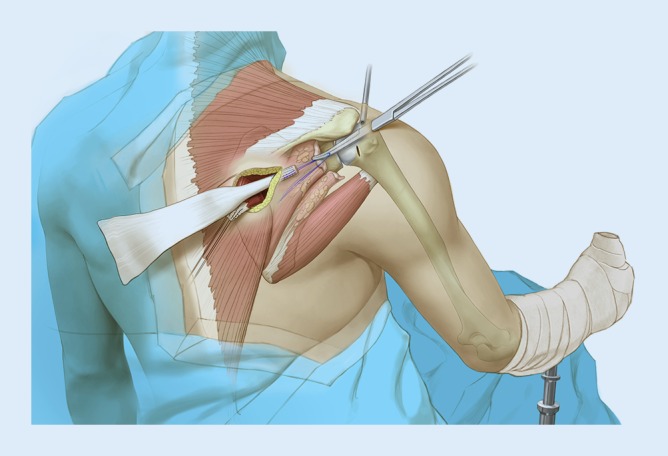
Schematic drawing demonstrating the passage of the Achilles tendon autograft through the lateral arthroscopic portal. (Used with permission of the Mayo Foundation for Medical Education and Research. All rights reserved)

An arthroscopically assisted technique has been described for a lower trapezius tendon transfer with Achilles tendon allograft augmentation ([6]; Fig. 4). The main purpose was to avoid violating the acromion and deltoid for exposure, and thus preventing nonunion of the acromial osteotomy. Additionally, arthroscopy improves visualization of the subscapularis tendon and facilitates repair.

The senior author (Bassem T. Elhassan) has performed an arthroscopically assisted lower trapezius tendon transfer on 41 patients. In a recent retrospective review (currently pending publication), at a mean follow-up of 13 months, 37 (90%) patients were found to have improvements in all outcome measures. Pseudoparalysis was reversed in more than 90% of patients. Reparable subscapularis tears did not affect outcomes. Degenerative changes, however, particularly Hamada stage 2 and 3 changes, led to worse outcomes in regard to pain and range of motion. Thus, lower trapezius tendon transfer in patients with advanced degenerative changes should be avoided or at least patients should be counselled on the likely risk of persistent pain and need for further surgery, such as a reverse total shoulder arthroplasty.

## Practical conclusion

- Lower trapezius tendon transfer with Achilles tendon allograft augmentation is an effective treatment for restoring active external rotation in patients with paralytic shoulders or irreparable posterosuperior rotator cuff tears.
- The procedure may be performed via an open technique or through an arthroscopically assisted procedure.
- For the procedure to be effective, surgeons should understand the principles and tenets of tendon transfer surgery as well as the biomechanics of glenohumeral articulation and rotator cuff force couples.
- For paralytic shoulders, careful evaluation of the integrity of the ipsilateral trapezius musculature is necessary. If compromised, the contralateral trapezius may be used.
- For irreparable rotator cuff tears, degenerative changes involving the glenohumeral joint must be evaluated on plain radiographs or magnetic resonance images. Advanced degenerative changes should prompt surgeons to recommend other treatment options, e.g., reverse total shoulder arthroplasty.
